# Evaluation of the Bactericidal and Fungicidal Activities of Poly([2-(methacryloyloxy)ethyl]trimethyl Ammonium Chloride)(Poly (METAC))-Based Materials

**DOI:** 10.3390/polym10090947

**Published:** 2018-08-26

**Authors:** Toshiki Shiga, Hiromitsu Mori, Keiichi Uemura, Ryota Moriuchi, Hideo Dohra, Aika Yamawaki-Ogata, Yuji Narita, Akihiro Saito, Yohei Kotsuchibashi

**Affiliations:** 1 Department of Materials and Life Science, Shizuoka Institute of Science and Technology, 2200-2 Toyosawa, Fukuroi, Shizuoka 437-8555, Japan; giants_421_10@yahoo.co.jp (T.S.); 1822008.mh@sist.ac.jp (H.M.); saito.akihiro@sist.ac.jp (A.S.); 2 Chutoen-General Medical Center, 1-1 Shobugaike, Kakegawa, Shizuoka 436-8555, Japan; k.uemura@fsinet.or.jp; 3 Research Institute of Green Science and Technology, Shizuoka University, 836 Ohya, Suruga-ku, Shizuoka City, Shizuoka 422-8529, Japan; moriuchi.ryota@shizuoka.ac.jp (R.M.); dora.hideo@shizuoka.ac.jp (H.D.); 4 Department of Cardiac Surgery, Nagoya University Graduate School of Medicine, 65 Tsurumai-cho, Showa-ku, Nagoya, Aichi 466-8550, Japan; aika@med.nagoya-u.ac.jp (A.Y.-O.); ynarita@med.nagoya-u.ac.jp (Y.N.)

**Keywords:** antimicrobial polymer, cationic polymer, poly([2-(methacryloyloxy)ethyl]trimethyl ammonium chloride), drug-resistant bacteria

## Abstract

Poly([2-(methacryloyloxy)ethyl]trimethyl ammonium chloride) (METAC) and the gels were prepared and evaluated for their bactericidal and fungicidal activities. The antimicrobial properties of poly(METAC) were tested against *Escherichia coli* (*E. coli*), *Bacillus subtilis* (*B. subtilis*), *Saccharomyces cerevisiae* (*Sa. cerevisiae*), methicillin-susceptible *Staphylococcus aureus* (MSSA), methicillin-resistant *Staphylococcus aureus* (MRSA), *Pseudomonas aeruginosa* (*P. aeruginosa*), and *Candida albicans* (*C. albicans*). Moreover, the structural forms of the linear and cross-linked poly(METAC) were investigated for their influences on bacterial aggregation, precipitation, and cell-death. To our knowledge, this is the first report on the comparison of the antimicrobial properties of poly(METAC) and poly(METAC)-gels. The bactericidal and fungicidal activities were evaluated by determining minimum inhibitory concentrations (MICs), UV–Vis spectroscopy, and fluorescence and confocal microscopies. The MICs were found to be 123 (MSSA), 123 (MRSA), 123 (*P. aeruginosa*), 370 (*E. coli*), 123 (*B. subtilis*), 370 (*C. albicans*), and 370 μg/mL (*Sa. cerevisiae*), as determined by broth dilution, and 370 (MSSA), 370 (MRSA), 370 (*P. aeruginosa*), 3300 (*E. coli*), 370 (*B. subtilis*), 1100 (*C. albicans*), and >10,000 μg/mL (*Sa. cerevisiae*), as determined by paper disc diffusion (on solid medium). The poly(METAC)-gels achieved rapid adsorption/precipitation of bacteria via the cationic surface charge. Thus, these poly(METAC)-based polymers can potentially be used as antibacterial materials.

## 1. Introduction

**Figure 1:**
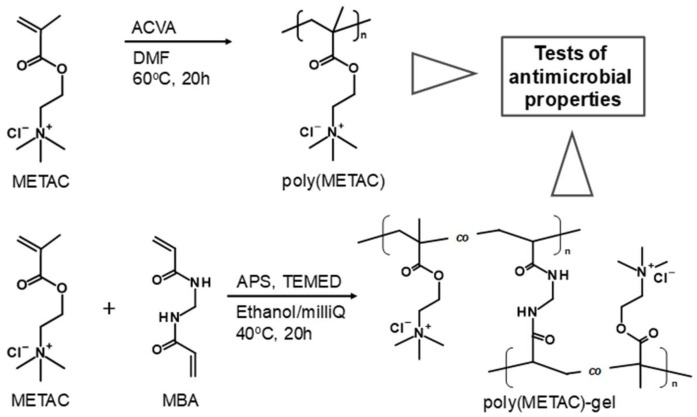
Synthesis of poly(METAC) and poly(METAC)-gel polymerized by a free radical polymerization.

Antimicrobial polymers have been receiving substantial attention owing to their multiple advantages, such as long-term activity, limited residual toxicity, chemical stability, non-volatility, and non-penetration via skin [1]. Cationic polymers are one of the most studied antimicrobial polymers [2,3]. The accepted mechanism by which cationic polymers lead to bacterial death is as follows: 1. adsorption onto the bacterial cell surface, 2. diffusion through the cell wall, 3. binding to the cytoplasmic membrane, and 4. disruption of the bacterial membrane [4]. This mechanism is effective regardless of the types of bacteria. Therefore, it is expected that the cationic polymers can also show an antimicrobial effect against drug-resistant bacteria. Recently, the World Health Organization (WHO) has listed the drug-resistant bacteria that could shortly become a menace to humans [5]. For example, in the US, the methicillin-resistant *Staphylococcus aureus* (MRSA) causes more deaths than human immunodeficiency virus (HIV) [6]. Very recently, Su et al. prepared cationic polypeptide-based polymers that present antimicrobial properties when administered in MRSA-infected mice [7,8]. The poly(hexamethylene biguanide), another type of cationic polymer, is approved by the Food and Drug Administration (FDA), and there are no reports on emergent drug-resistant bacteria [9,10]. Poly([2-(methacryloyloxy)ethyl]trimethylammonium chloride) (poly(METAC)) is one of the methacrylate types of cationic polymer, consisting of quaternary ammonium cations. The methacrylate monomer can be polymerized by (living/controlled) radical polymerizations and can also be copolymerized with other types of typical (meth)acrylate monomers [11,12,13]. The development of polymeric synthesis has facilitated access to functional polymers, allowing not only the control of molecular weights and molecular weight distribution, but also the control of the physicochemical properties of the polymeric structures [14,15]. Stopiglia et al. showed that the METAC monomer presents antimicrobial properties against 31 kinds of *Candida albicans* (*C. albicans*) [16]. Prijick et al. succeeded in preventing the formation of a *C. albicans* biofilm on a surface coated with copolymers of poly(METAC) [17]. The poly(METAC) has also been conjugated with natural polymers. The cotton grafting poly(METAC) shows antimicrobial properties against Gram-positive and Gram-negative bacteria, and this effect increases with the amount of grafted poly(METAC) [18]. A wool-modified surface was combined with the chitosan grafting poly(METAC), and the conjugated materials shows efficient antimicrobial properties [19]. In this study, the antimicrobial properties of poly(METAC) were tested against *Escherichia coli* (*E. coli*), *Bacillus subtilis* (*B. subtilis*), *Saccharomyces cerevisiae* (*Sa. cerevisiae*), methicillin-susceptible *Staphylococcus aureus* (MSSA), MRSA, *Pseudomonas aeruginosa* (*P. aeruginosa*), and *C. albicans*. In addition, poly(METAC)-gel was prepared to allow a comparison of the two different structural forms, linear and cross-linked poly(METAC), on their ability to induce bacterial aggregation, precipitation, and cell-death. To our knowledge, this is the first report on a comparison of antimicrobial properties of poly(METAC) and poly(METAC)-gel (Figure 1). The minimum inhibitory concentration (MIC) was determined by broth dilution and paper disc diffusion methods, and the bacterial aggregation, precipitation, and cell-death were measured by UV–Vis spectroscopy, and fluorescence and confocal microscopies.

## 2. Materials and Methods

### 2.1. Materials

[2-(methacryloyloxy)ethyl]trimethylammonium chloride (METAC) (80 wt % in water), 4,4′-azobis(4-cyanovaleric acid) (ACVA), and *N*,*N*′-methylenebis(acrylamide) (MBA) were purchased from Sigma-Aldrich (St Louis, MO, USA) and were used as received. All other chemicals and solvents were used as received. *Escherichia coli* JM109 [20], *Bacillus subtillis* 168, and a strain of *Saccharomyces cerevisiae* isolated from commercially obtained dry yeast were (Nisshin Seifun Group Inc., Tokyo, Japan) used as representatives for Gram-negative, Gram-positive bacteria, and fungi *B. subtilis* 168 (JCM 10629) was obtained from the Japan Collection of Microorganisms (JCM, Ibaraki, Japan), BioResource Research Center, RIKEN, Ibaraki, Japan. *Pseudomonas aeruginosa* NBRC 12689^T^ (=ATCC 10145) and *Candida albicans* NBRC 1385^T^ (=ATCC 18804) were supplied from the Biological Resource Center, NITE (NBRC), Tokyo, Japan. Methicillin-susceptible strain KUSA01 and a resistant strain KUSA02 of *Staphylococcus aureus* used in this study were clinically isolated.

### 2.2. Preparation of Poly(METAC) and Poly(METAC)-gel

Free radical polymerization was employed to synthesize poly(METAC) and poly(METAC)-gel. Poly(METAC) was prepared as follows. METAC (2.60 g (including water), 10 mmol) and ACVA (14.0 mg, 0.050 mmol) ([METAC]_0_/[ACVA]_0_ = 200/1) were dissolved in 5 mL of *N*,*N*-dimethylformamide (DMF). After degassing with nitrogen gas for 30 min, polymerization was allowed for 24 h at 60 °C. The resulting poly(METAC) was purified by dialysis, firstly against ethanol/water 1/1 (*v*/*v*) and then water, and dried by lyophilization. Poly(METAC)-gel was prepared using different amounts of a cross-linker of *N*,*N*′-methylenebis(acrylamide) (MBA). METAC (2.60 g (including water), 10 mmol) and MBA (10, 20, 30, or 50 mg; 0.065, 0.13, 0.19, or 0.32 mmol) were dissolved in 10 mL of a mixture solution of ethanol/water 1/1 (*v*/*v*) at 40 °C. Two-hundred microliters of ammonium peroxodisulfate (APS) solution (10 wt % in water) and 20 μL of *N*,*N*,*N*′,*N*′-Tetramethylethylenediamine (TEMED) were added to the mixture solution, and this was incubated for 20 h at 40 °C, to allow polymerization. The resulting poly(METAC)-gel was purified via immersion, firstly in ethanol/water 1/1 (*v*/*v*), and then in ethanol. After the purification, the poly(METAC)-gel was dried under reduced pressure. Poly(METAC)-gels were labeled as the poly(METAC)-gel-X, in which X is the mol % of cross-linker in the total of METAC monomer and cross-linker (i.e., X = 0.65, 1.28, 1.86, and 3.10). For example, the 0.65 was calculated via the (0.065/(10 + 0.065)) × 100.

### 2.3. Determination of Minimum Inhibitory Concentration (MIC) of Poly(METAC)

MICs were determined by broth dilution and paper disc diffusion methods. Luria broth was used for *E. coli* and *B. subtillis*, Müller Hinton broth (MHB) for *S. aureus*, cation-adjusted MHB for *P. aeruginosa*, potato-dextrose broth for *S. cerevisiae*, and MHB containing glucose (2% (*w*/*v*)) and methylene blue (0.5 μg/mg) for *C. albicans*. Except for *P. aeruginosa*, corresponding agar media were used for the paper disc diffusion method. Müller Hinton agar medium was used for *P. aeruginosa*. The suitable broths for *S. aureus*, *P. aeruginosa*, and *C. albicans* were selected using the performance standards for antimicrobial susceptibility testing of the medical field. Other bacteria were incubated using the typical broths. Bacterial suspensions were prepared via the turbidity (McFarland standards) in accordance with each bacterial performance standards. For the broth dilution method, microbial cells (150 μL of the overnight culture fluid) cultivated overnight at 37 °C were inoculated into fresh liquid medium supplemented with poly(METAC) and subsequently incubated for 24 h at 37 °C with shaking at 150 rpm. MIC was defined as the minimum poly(METAC) concentration that completely inhibited microbial growth. For the paper disc diffusion method, microbial cells were spread onto agar medium, over which a series of discs containing 0–1.0% (*w*/*v*) poly(METAC) were placed. Cells were cultivated for 24 h at 37 °C and growth of microorganisms around the discs were observed. MIC was defined as the minimum poly(METAC) concentration in discs leading to growth inhibition zones. 

### 2.4. Evaluation of Bacterial Aggregation/Precipitation by Poly(METAC) and Poly(METAC)-gel

*B. subtilis* cells grown in LB liquid medium until the mid-exponential phase were exposed to poly(METAC) or poly(METAC)-gel-1.28 at different concentrations (1–5 mg/1.5 mL) for 24 h. The appropriate amounts of poly(METAC) or poly(METAC)-gel-1.28) were dissolved/dispersed in 500 μL milliQ and mixed with *B. subtilis* suspension (1 mL). After 24 h, the transmittance of the supernatants was measured using UV–Vis spectroscopy at 500 nm.

### 2.5. Evaluation of the Bactericidal Effect of Poly(METAC) and Poly(METAC)-gel

*E. coli* cells grown in LB liquid medium until the mid-exponential phase were exposed to poly(METAC) or MilliQ water (as control) and immediately subjected for the next procedures. To evaluate the bactericidal activity of the polymer, cells treated with poly(METAC) were stained with SYTO9 and propidium iodide (PI) using the LIVE/DEAD BacLight Bacterial Viability Kit (Life Technologies, Carlsbad, CA, USA), by following the manufacturer’s instructions, and observed under a fluorescence microscope. To evaluate bacterial survival in the presence of poly(METAC), *E. coli* cells exposed to different concentrations of poly(METAC) were washed once with phosphate buffered saline [21], spread on LB agar medium, and incubated at 37 °C for 24 h, after which the colonies were counted. Adsorbed bacteria on poly(METAC)-gel were observed by confocal microscopy. The poly(METAC)-gels were immersed into an *E. coli* suspension for 10 min and washed 2 times with large amounts of pH 7.4 phosphate-buffered saline (PBS). Bacterial cells on the poly(METAC)-gels were stained using the LIVE/DEAD BacLight Bacterial Viability Kit.

### 2.6. Characterizations

Number-average molecular weight (*M*_n_) and molecular weight distribution (*M*_w_/*M*_n_, *M*_w_: weight-average molecular weight) of the synthesized poly(METAC) were determined by gel permeation chromatography (GPC) (Shimadzu, Kyoto, Japan) at room temperature, with SB-802.5 HQ and SB-804 HQ columns (Shodex, Tokyo, Japan) connected to a RID-20A refractive index detector (Shimadzu, Kyoto, Japan). Elution was performed with 0.5 M sodium acetate/0.5 M acetic acid buffer at a flow rate of 1.0 mL/min. Transmittance of mixture suspensions of bacteria and polymeric materials at 500 nm was recorded at 25 °C on a UV–Vis spectrometer V-650 (JASCO International Co., Ltd., Tokyo, Japan). A microplate reader Infinite M1000-SSY (TECAN, Kanagawa, Japan), a fluorescence microscope MX (MEIJI Techno, Saitama, Japan) with an excitation light source FL-PWJ (MEIJI Techno, Saitama, Japan), and a confocal microscope LSM 700 (Carl Zeiss, Oberkochen, Germany) were used to evaluate the bactericidal effect of poly(METAC) copolymers.

## 3. Results

Molecular weight (*M*_n_) and molecular weight distribution (*M*_w_/*M*_n_) of poly(METAC) were determined to be 87,400 g/mol and 2.83, respectively (measured by GPC). The synthesized poly(METAC) inhibited the growth of all the tested microorganisms: Gram-positive bacteria (*S. aureus* and *B. subtilis*), Gram-negative bacteria (*P. aeruginosa* and *E. coli*), and yeasts (*C. albicans* and *Ss. cerevisiae*). The determined minimum inhibitory concentrations (MICs) for these microorganisms are shown in Table 1. The MICs determined by broth dilution method (in liquid media) were found to be 123 μg/mL (MSSA), 123 μg/mL (MRSA), 123 μg/mL (*P. aeruginosa*), 370 μg/mL (*E. coli*), 123 μg/mL (*B. subtilis*), 370 μg/mL (*C. albicans*), and 370 μg/mL (*Sa. cerevisiae*). The MICs determined by paper disc diffusion method (on solid media) were found to be 370 μg/mL (MSSA), 370 μg/mL (MRSA), 370 μg/mL (*P. aeruginosa*), 3300 μg/mL (*E. coli*), 370 μg/mL (*B. subtilis*), 1100 μg/mL (*C. albicans*), and >10,000 μg/mL (*Sa. cerevisiae*). The MICs of broth dilution method were lower than that of paper disc diffusion method, for all the microorganisms. These results suggested that the poly(METAC) exhibits antibacterial properties against Gram-positive bacteria, Gram-negative bacteria, and yeasts. Moreover, it presents similar antibacterial levels against both of MSSA and MRSA. Rawlinson et al. reported resistance of *S. aureus* to a cationic polymer of poly(2-(dimethylamino ethyl)methacrylate)(poly(DMAEMA)), when compared with *S. epidermidis*. The resistance was explained as resulting from the low negative charge and hydrophobicity of the *S. aureus* surface [22]. In fact, the MICs of every *S. aureus* strain (clinical isolate species, two MSSA and six MRSA) were all over 3200 μg /mL. On the other hand, the MICs of eleven types of *S. epidermidis* were in the range 25–100 μg/mL. Moreover, the adhesion of poly(DMAEMA) to *S. aureus* was lower than that of *S. epidermidis*, as measured by flow cytometry. The polymeric structures also affect the antimicrobial and adsorptive properties on the target bacteria. Shirbin et al. prepared a macroporous cryogel using cationic polypeptide of poly(lysine)-*b*-poly(valine), and the cryogel showed a “trap and kill” effect against *E. coli* [23]. The cationic four-arm star glycopolymer-peptides showed different MICs depending on the glycol-types such as glucose, galactose, or mannose (*E. coli*: 10–256 μg/mL, *P. aeruginosa*: 32–512 μg/mL, and *S. aureus* 16–128 μg/mL) [24].

**Figure 2:**
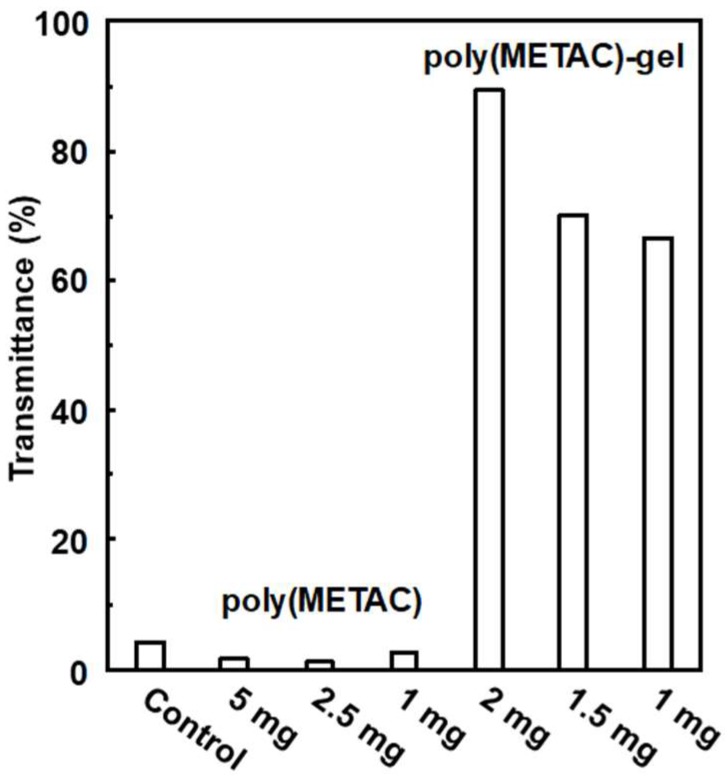
Transmittance measurement of the suspensions of poly(METAC) or poly(METAC)-gel-1.28 with *B. subtilis*. The *B. subtilis suspensions* were exposed to poly(METAC) or poly(METAC)-gel-1.28 at different concentrations for 24 h. The total amount of solution was 1.5 mL. After 24 h, the transmittances of supernatants were measured at 500 nm.

Next, poly(METAC)-gels were tested on their abilities to induce bacterial aggregation, precipitation, and cell-death. The poly(METAC)-gels-1.28 were prepared via redox polymerization using *N*,*N*′-methylenebis(acrylamide) as a cross-linker [25]. Figure 2 shows the transmittance changes of *B. subtilis* suspensions treated by poly(METAC) or poly(METAC)-gel-1.28 after 24 h. The control showed that less than 5% of the observed transmittances were due to the suspended *B. subtilis*. Similarly, less than 5% of the observed transmittances of the *B. subtilis* suspensions were due to the adding of the poly(METAC) solution, which were similar to control. On the other hand, the transmittance tended to increase with the amount of poly(METAC)-gel-1.28, and reached 90% with 2 mg of the gel. The weak bacterial aggregation/precipitation in the presence of poly(METAC) is caused by the electrostatic repulsion of the adsorbing poly(METAC) on the bacterial surface. The bacteria trapped on the poly(METAC)-gel-1.28 cannot be re-suspended via the electrostatic interaction between the anionic bacterial surface and cationic gel surface, resulting in the high transmittance values. In the nonionic poly(2-hydroxyethyl methacrylate (HEMA))-gel, the transmittance was lower than 5% (Figure S1). The interaction of nonionic poly(HEMA)-gels with bacteria was smaller than that of the cationic polymers. Similar results were reported by Berlutti’s group [26]. Williams et al. prepared poly(METAC)-based cationic temperature-responsive gels, which show bacteriostatic and bactericidal properties against *S. aureus* when compared with that of nonionic gels [27]. 

**Figure 3:**
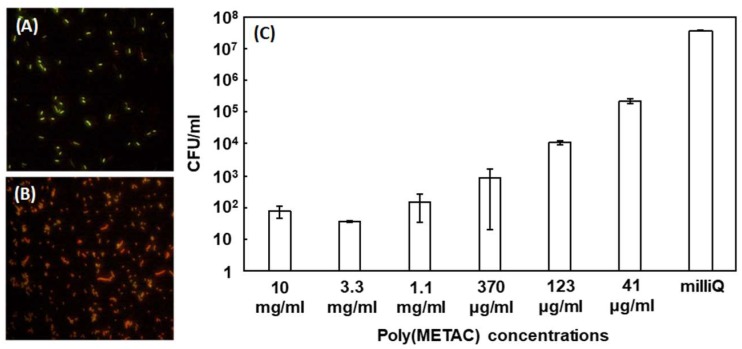
LIVE/DEAD test of *E. coli* in (**A**) milliQ and (**B**) 1 wt % of poly(METAC). The *E. coli* were stained with SYTO9 and PI. (**C**) The survival test of *E. coli* in the presence of poly(METAC) at different concentrations. The exposed *E. coli* by poly(METAC) were washed using phosphate-buffered saline (PBS) and were spread on medium to count the number of survived *E. coli* cells.

**Figure 4:**
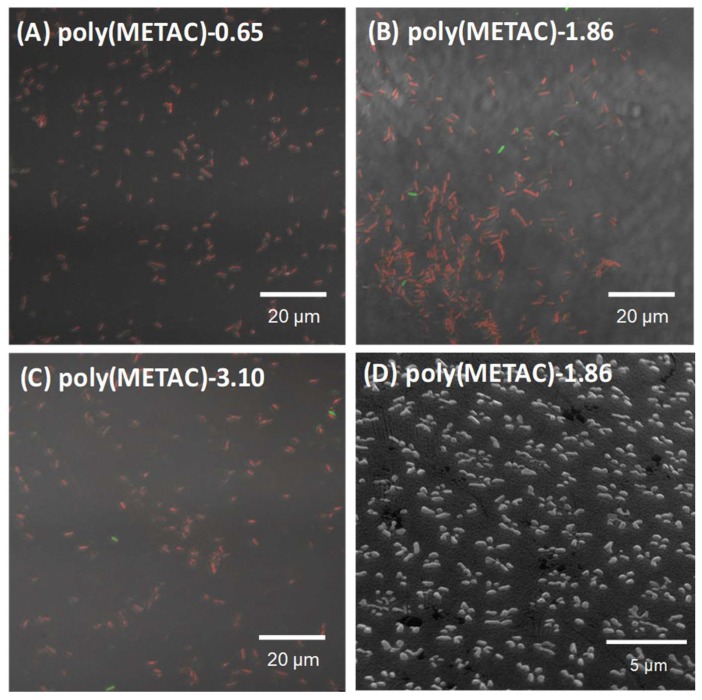
Confocal images of LIVE/DEAD test of *E. coli* on poly(METAC)-gels at various crosslinker amount; (**A**) 0.65 mol %, (**B**) 1.86 mol %, and (**C**) 3.10 mol %. The *E. coli* were stained with SYTO9 and PI. (**D**) Scanning electron microscope (SEM) images of the adsorbing *E. coli* on poly(METAC)-gels-1.86.

Next, the biocidal ability of the poly(METAC) and poly(METAC)-gels was investigated. Figure 3 shows the LIVE/DEAD test of *E. coli* in the presence of MilliQ (Figure 3A) or 1 wt % of poly(METAC) (Figure 3B). The *E. coli* were stained with SYTO9/PI, which leads to the exhibition of red fluorescence by the death cells due to the treatment with poly(METAC). In another experiment, *E. coli* were exposed to poly(METAC) at different concentrations (10 mg/mL–41 μg/mL), spread on the nutrient agar, and the colony forming units (CFUs) were measured after 24 h. Figure 3C shows the CFU at each poly(METAC) concentration. CFUs decreased with increasing poly(METAC) concentration, and reached a plateau (about 100 CFU/mL) at over 1.1 mg/mL. The prevention of the *E. coli* growth by poly(METAC) was about 0.4 million-fold higher than that of the control without poly(METAC). Adsorbed bacteria on poly(METAC)-gels were observed by the confocal microscopy. The poly(METAC)-gels were immersed in the *E. coli* suspension for 10 min, and were washed 2 times with large amounts of pH 7.4 PBS. The bacteria on the poly(METAC)-gels were stained with the live/dead agent. Figure 4A–C shows confocal microscopy images of the poly(METAC)-gels with different cross-linker amounts, 0.65, 1.86, and 3.10 mol %. Almost all bacteria on the poly(METAC)-gels were dead as shown by the red fluorescence. The adsorbed bacteria were also observed by scanning electron microscopy (SEM) measurement (Figure 4D). He et al. prepared a cationic hydrogel film via a light-triggered cross-linking [28]. The antimicrobial activity against *E. coli* was shown to depend on the amount of the ethylene glycol-based cross-linkers. At 20% of cross-linking degree, approximately 100% of antibacterial activity was reached. Yang et al. prepared a hydrogel consisting of METAC and nonionic monomers bound by disulfide bonds, and the gels successfully prevented the adsorption of proteins and *E. coli* on the surface [29]. The physical destruction of the adsorbing *E. coli* on the gel surface was observed via SEM measurement. Other nonionic polymers such as ethylene glycol-based copolymers also can prevent the adsorption of bacteria [30,31]. The METAC is a methacrylate type of monomer and the polymeric structure can be easily controlled via a combination of living radical polymerizations and click chemistry. The antibacterial properties will be customized by the structural formulation of poly(METAC).

## 4. Conclusions

In conclusion, poly([2-(methacryloyloxy)ethyl]trimethyl ammonium chloride) (METAC) and poly(METAC)-gels were evaluated for their bactericidal and fungicidal activities. The MICs were found to be 123 (MSSA), 123 (MRSA), 123 (*P. aeruginosa*), 370 (*E. coli*), 123 (*B. subtilis*), 370 (*C. albicans*), and 370 μg/mL (*Sa. cerevisiae*) as determined by broth dilution. And, the MICs measured by paper disc diffusion method (on solid media) were 370 (MSSA), 370 (MRSA), 370 (*P. aeruginosa*), 3300 (*E. coli*), 370 (*B. subtilis*), 1100 (*C. albicans*), and >10,000 μg/mL (*Sa. cerevisiae*). Importantly, poly(METAC) showed similar levels of antibacterial activity against both MSSA and MRSA. The poly(METAC)-gels showed rapid adsorption/precipitation of bacteria, with almost all bacteria being killed via the adsorption on poly(METAC)-gels within 10 min. The METAC is a relatively low-cost monomer and can be copolymerized with typical (meth)acrylate and (meth)acrylamide monomers via the living radical polymerizations. Moreover, it is also easy to combine with the existing water-soluble polymers, such as poly(vinyl alcohol), poly(ethylene glycol), alginic acid, etc. The antibacterial properties will be customized by the structural formulation of poly(METAC), and the properties will be applied in the antibacterial dressing materials.

Thus, these poly(METAC)-based copolymers can potentially be used as antibacterial materials.

## Figures and Tables

**Table 1:** Minimum inhibitory concentrations (MICs) of poly(METAC) synthesized in this study.

		MIC (μg/mL) *	
		in Liquid Media	on Solid Media
*S. aureus*	MS	123	370
	MR	123	370
*P. aeruginosa*		123	370
*E. coli*		370	3300
*B. subtilis*		123	370
*C. albicans*		370	1100
*Sa. cerevisiae*		370	10,000<

* MICs were determined by broth dilution method (in liquid media) and paper disc diffusion method (on solid media). Please see Materials and Methods for detailed experimental conditions. *S. aureus*, *Staphylococcus aureus*; *P. aeruginosa*, *Pseudomonas aeruginosa*; *E. coli*, *Escherichia coli*; *B. subtilis*, *Bacillus subtilis*; *C. albicans*, *Candida albicans*; *Sa. cerevisiae*, *Saccharomyces cerevisiae*; MS, methicillin-susceptible strain; MR, methicillin-resistant strain.

## Supplementary Materials

The following are available online at http://www.mdpi.com/2073-4360/10/9/947/s1, Figure S1: Transmittance measurement of the suspensions of poly(METAC)-gel with *B. subtilis*.
