# Supplementary material for: Evaluation of the Bactericidal and Fungicidal Activities of Poly([2-(methacryloyloxy)ethyl]trimethyl Ammonium Chloride)(Poly (METAC))-Based Materials
Source: Polymers (Basel). 2018 Aug 26;10(9):947. doi: 10.3390/polym10090947 (PMC6403553; doi:10.3390/polym10090947)
Supplement: Supplementary file 1 [file polymers-10-00947-s001.pdf]

Supporting Information

Evaluation of the bactericidal and fungicidal activities of poly([2-(methacryloyloxy)ethyl]trimethyl ammonium chloride)(poly(METAC))-based materials

Toshiki Shiga [1], Hiromitsu Mori [1], Keiichi Uemura [2], Ryota Moriuchi [3], Hideo Dohra [3], Aika Yamawaki-Ogata [4], Yuji Narita [4], Akihiro Saito [1], and Yohei Kotsuchibashi [1]

[1]Department of Materials and Life Science, Shizuoka Institute of Science and Technology, 2200-2 Toyosawa, Fukuroi, Shizuoka 437-8555, Japan; giants_421_10@yahoo.co.jp (T.S.); 1822008.mh@sist.ac.jp (H.M.); saito.akihiro@sist.ac.jp (A.S.)

[2]Chutoen-General Medical Center, 1-1 Shobugaike, Kakegawa, Shizuoka 436-8555, Japan; k.uemura@fsinet.or.jp (K.U.)

[3]Research Institute of Green Science and Technology, Shizuoka University, 836 Ohya, Suruga-ku, Shizuoka city, Shizuoka 422-8529, Japan; moriuchi.ryota@shizuoka.ac.jp (R.M.); dora.hideo@shizuoka.ac.jp (H.D.)

[4]Department of Cardiac Surgery, Nagoya University Graduate School of Medicine, 65 Tsurumai-cho, Showa-ku, Nagoya, Aichi, 466-8550, Japan; aika@med.nagoya-u.ac.jp (A.Y.-O.); ynarita@med.nagoya-u.ac.jp (Y.N.)

*Correspondence: kotsuchibashi.yohei@sist.ac.jp (Y.K.); Tel.: +81-538-45-0111

Preparation of poly(2-hydroxyethyl methacrylate)-gel (poly(HEMA)-gel)

HEMA (1.30 g, 10 mmol) and *N*,*N*′-methylenebis(acrylamide) (MBA) (20 mg, 0.13 mmol) were dissolved in 10 mL of water. A 200 μL of ammonium peroxodisulfate (APS) solution (10 wt% in water) and 20 μL of *N*,*N*,*N*′,*N*′-Tetramethylethylenediamine (TEMED) were added to the mixture solution. The solution was allowed to polymerize for 20 h at 25 °C. The resulting P(HEMA)-gel was purified via immersion against to water. After the purification, the P(HEMA)-gel was dried under reduced pressure.

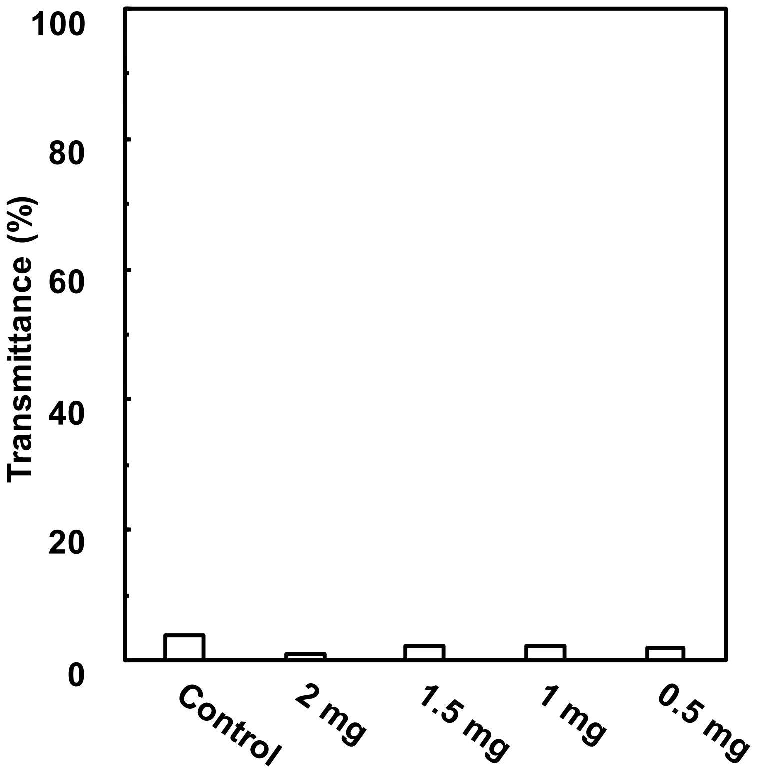

Figure S1. Transmittance measurement of the suspensions of poly(METAC)-gel with *B. subtilis*. The *B. subtilis suspensions* were exposed to poly(METAC)-gel at different concentrations for 24 h. The total amount of solution was 1.5 mL. After 24 h, the transmittances of supernatants were measured at 500 nm.
